# Evaluation of potential genetic marker for growth and carcass traits in Sumba Ongole (*Bos indicus*) cattle

**DOI:** 10.5455/javar.2024.k751

**Published:** 2024-03-31

**Authors:** Paskah Partogi Agung, Ferdy Saputra, Widya Pintaka Bayu Putra, Syahruddin Said, Moch. Syamsul Arifin Zein, Febrina Hastuti Harianja, Aditya Sudiro

**Affiliations:** 1 Research Center for Applied Zoology-BRIN, Bogor, Indonesia; 2 Research Center for Animal Husbandry-BRIN, Bogor, Indonesia; 3 Research Center for Biosystematics and Evolution-BRIN, Bogor, Indonesia; 4 Center for Quality Testing and Certification of Veterinary Medicines (BBPMSOH), Bogor, Indonesia; 5 PT. Karya Anugerah Rumpin (PT. KAR), Bogor, Indonesia

**Keywords:** Carcass, growth, marker, polymorphism, Sumba Ongole

## Abstract

**Objective:**

This study was conducted to investigate the variants of the growth hormone receptor (GHR), growth hormone-releasing hormone (GHRH), pituitary-specific transcription factor-1 (PIT1), and signal transducer and activator of transcription 5A (STAT5) genes and their effect on growth performance and dressing percentage (DP) parameters.

**Materials and Methods:**

A total of 401 DNA samples from Sumba Ongole (SO) cattle were utilized for the polymerase chain reaction-restriction fragment length polymorphism method, of which 200 samples were used for the study of DP association and 74 samples were used to investigate growth performance. The SO cattle growth performance includes the following: birth weight, weaning weight at 205 days of age, weaning average daily gain (ADG), yearling weight at 365 days of age, and post-weaning ADG.

**Results:**

The GHR, GHRH, PIT1, and STAT5A genes showed polymorphism. The highest polymorphism information content value was shown in the STAT5A gene. The highest DP value was found in the SO cattle population with the CC genotype (STAT5A), and the lowest DP value was found in the SO cattle population with the GG genotype (GHR). The GHR and STAT5A genotypes were highly associated (*p* < 0.05) with the DP parameter. Based on locus combination analysis, the highest DP value was found in the SO cattle population with AG|CC genotype (GHR|STAT5A) (57.85%), AG|BB|CC genotype (GHR|GHRH|STAT5A) (57.85%), and AA|BB|BB|CC genotype 18 (GHR|GHRH|PIT1|STAT5A) (56.02%).

**Conclusion:**

All investigated genes in this study were polymorphic but were not associated with several growth parameters. The GHR and STAT5A genes can be proposed as genetic markers for the high DP trait in SO cattle in Indonesia, especially the AA genotype (GHR) and CC genotype (STAT5A).

## Introduction

Animal growth parameters are important in livestock breeding programs. Some hormones that are associated with animal growth have been reported, including growth hormone receptor (GHR) [1], growth hormone-releasing hormone (GHRH) [2], pituitary-specific transcription factor-1 (PIT-1) [3], and signal transducer and activator of transcription 5A (STAT5A) [4]. The GHR acts as a transmembrane protein, and its primary function is to bind GH with great specificity and affinity. The cattle GHR gene contains nine exons [5]. GHRH affects growth, and different metabolic processes [6] and is linked to several growth indicators [7].

The PIT1 gene was considered a possible genetic marker in cattle breeding due to its correlation with growth traits [8]. The STAT proteins regulate cytokine signaling pathways and gene transcription. STAT5A and STAT5B differ based on the total number of amino acids and are also encoded by different genes. The STAT5A gene was proposed as a genetic marker to increase milk production and fertility in Holstein cattle [9].

Up to the present, the selection of Sumba Ongole (SO) cattle in Indonesia still uses traditional methods or a breeding value scheme without additional genetic information [10]. The genetic information of the SO cattle breed in Indonesia is still limited. Several previous studies revealed that the IGF-1, Calpain, and MC4R genes were not recommended as genetic markers in SO cattle [11–13]. Hence, investigation of another gene is important to establish a breeding scheme for the SO cattle, especially to generate a superior SO cattle population using genetic markers (marker-assisted selection). This study aims to investigate the variety of the GHR, GHRH, PIT1, and STAT5A genes in SO cattle and also evaluate their effect on growth performance indicators and dressing percentage (DP) parameters.

## Materials and Methods

This study was conducted under the ethical approval of the Indonesian Institute of Sciences (Register No. 9879/WK/HK/XI/2015).

The genotyping analysis was conducted using 401 DNA samples, consisting of 74 samples that have information on growth data, 200 samples that have information on carcass yield, and 127 samples that did not have growth data or carcass yield information but were included in the genotyping and allele frequency calculation. The growth data, i.e., birth weight (BW), weaning weight at 205 days of age (WW205), weaning average daily gain (ADG^1^), yearling weight at 365 days of age (YW365), and post-weaning ADG2, were collected from Karya Anugerah Rumpin Corporation (PT. KAR) breeding farm in Bogor, Indonesia. The animals were fed based on the energy recommendations for calves. The carcass yield data were collected from two animal slaughterhouses, i.e., the PT. KAR slaughterhouse in Banten Province and the East Sumba slaughterhouse in East Nusa Tenggara Province. The carcass yield data was converted into DP data.

**Table 1.** The allele and genotype frequencies of the loci in the SO cattle.

Locus	Genotype (*n*) frequency			Allele frequency		H_o_	H_e_	PIC
GHR	AA (308)	AG (84)	GG (9)	A	G	0.21	0.22	0.20
	0.77	0.21	0.02	0.87	0.13			
GHRH	AA (11)	AB (123)	BB (267)	A	B	0.31	0.30	0.25
	0.03	0.31	0.66	0.18	0.82			
PIT1	AA (4)	AB (60)	BB (337)	A	B	0.15	0.16	0.14
	0.01	0.15	0.84	0.08	0.92			
STAT5A	CC (22)	CT (370)	TT (9)	C	T	0.92	0.50	0.38
	0.06	0.92	0.02	0.52	0.48			

*n =* individuals genotyped; H_e_=expected heterozygosity; H_o_=observed heterozygosity; PIC=polymorphism information content.

**Table 2.** Descriptive statistics for the investigated growth traits in the SO cattle.

Locus	Genotype (*n*)	BW	WW_205_	YW_365_	ADG^1^	ADG^2^
		-----------------------------Mean ± standard deviation------------------------------				
GHR	AA (54)	26.62 ± 5.85^a^	108.93 ± 27.93^a^	251.90 ± 104.60^a^	0.40 ± 0.14^a^	0.69 ± 0.29^a^
	AG (19)	24.09 ± 5.49^a^	110.07 ± 21.99^a^	223.50 ± 74.4^a^	0.42 ± 0.10^a^	0.61 ± 0.20^a^
	GG (1)*	27.00	149.60	167.0	0.60	0.46
GHRH	AA (1)*	25.00	114.40	189.00	0.44	0.52
	AB (20)	27.27 ± 4.29^a^	106.34 ± 29.91^a^	213.30 ± 98.50^a^	0.39 ± 0.15^a^	0.58 ± 0.27^a^
	BB (53)	25.50 ± 6.28^a^	110.98 ± 25.70^a^	255.90 ± 96.40^a^	0.42 ± 0.13^a^	0.70 ± 0.26^a^
PIT1	AA (1)*	16.80	62.83	463.98	0.22	1.27
	AB (10)	25.22 ± 4.03^a^	115.08 ± 26.97^a^	259.50 ± 129.50^a^	0.44 ± 0.14^a^	0.71 ± 0.36^a^
	BB (63)	26.24 ± 5.96^a^	109.67 ± 26.22^a^	237.40 ± 89.10^a^	0.41 ± 0.13^a^	0.65 ± 0.24^a^
STAT5	CC (2)	25.20 ± 6.79^a^	107.10 ± 35.20^a^	205.85 ± 10.12^a^	0.40 ± 0.14^a^	0.56 ± 0.03^a^
	CT (69)	25.92 ± 5.92^a^	109.75 ± 26.39^a^	246.90 ± 100.10^a^	0.41 ± 0.13^a^	0.68 ± 0.27^a^
	TT (3)	27.67 ± 2.08^a^	111.90 ± 39.00^a^	190.20 ± 32.70^a^	0.41 ± 0.20^a^	0.52 ± 0.09^a^

*n =* number of samples; BW=birth weight (kg); WW_205_=weaning weight at 205 days of age (kg); ADG^1^=weaning average daily gain (kg/day); YW_365_=yearling weight at 365 days of age (kg); ADG^2^=post weaning average daily gain (kg/day); different superscript letters differ significantly (*p* < 0.05); *=not included in statistical analysis.

**Table 3.** Statistical analysis result of growth traits in the SO cattle based on genotype combinations.

Combination	BW (kg)	WW_205_ (kg)	YW_365_ (kg)		ADG^1^ (kg)		ADG^2^ (kg)
	………………………………………..			Genotype (n)		………………………………	
**Mean ± standard deviation**	
GHR|GHRH	AA|AB (14) 28.50 ± 4.40	AG|BB (14) 113.33 ± 18.28	AG|BB (14) 238.10 ± 77.40		AG|BB (14) 0.43 ± 0.08		AA|BB (39) 0.72 ± 0.28
GHR|PIT1	AA|BB (45) 26.94 ± 6.03	AG|BB (17) 122.49 ± 20.18	AA|AB (8) 285.30 ± 131.70		AA|AB (8) 0.47 ± 0.12		AA|AB (8) 0.78 ± 0.36
GHR|STAT5A	AA|CT (51) 26.49 ± 5.98	AG|CT (18) 111.61 ± 21.54	AA|CT (51) 254.60 ± 106.90		AG|CT (18) 0.43 ± 0.10		AA|CT (51) 0.70 ± 0.29
GHRH|PIT1	AB|BB (17) 27.49 ± 4.59	BB|AB (7) 124.40 ± 17.47	BB|AB (7) 278.20 ± 120.80		BB|AB (7) 0.49 ± 0.10		BB|AB (7) 0.76 ± 0.33
GHRH|STAT5A	AB|CT (17) 27.76 ± 4.30	BB|CT (51) 111.33 ± 25.44	BB|CT (51) 257.20 ± 98.00		BB|CT (51) 0.42 ± 0.13		BB|CT (51) 0.70 ± 0.27
PIT1|STAT5A	BB|CT (58) 26.20 ± 6.13	AB|CT (10) 115.08 ± 26.97	AB|CT (10) 259.50 ± 129.50		AB|CT (10) 0.44 ± 0.14		AB|CT (10) 0.71 ± 0.36
GHR|GHRH|PIT1	AA|AB|BB (12) 28.75 ± 4.73	AA|BB|AB (6) 126.07 ± 18.52	AA|BB|AB (6) 292.30 ± 125.90		AA|BB|AB (6) 0.49 ± 0.11		AA|BB|AB (6) 0.80 ± 0.35
GHR|GHRH|STAT5A	AA|AB|CT (13) 28.69 ± 4.52	AG|BB|CT (14) 113.33 ± 18.28	AA|BB|CT (37) 264.50 ± 104.80		AG|BB|CT (14) 0.43 ± 0.08		AG|BB|CT (14) 0.65 ± 0.21
GHR|PIT1|STAT5A	AA|BB|CT (42) 26.81 ± 6.20	AA|AB|CT (8) 121.47 ± 22.91	AA|AB|CT (8) 285.30 ± 131.70		AA|AB|CT (8) 0.47 ± 0.13		AA|AB|CT (8) 0.78 ± 0.36
GHRH|PIT1|STAT5A	AB|BB|CT (14) 28.14 ± 4.62	BB|AB|CT (7) 124.40 ± 17.47	BB|AB|CT (7) 278.20 ± 120.80		BB|AB|CT (7) 0.49 ± 0.10		BB|AB|CT (7) 0.76 ± 0.33
GHR|GHRH|PIT1|STAT5A	AA|AB|BB|CT (11) 29.00 ± 4.88	AA|BB|AB|CT (6) 126.07 ± 18.52	AA|BB|AB|CT (6) 292.30 ± 125.90		AA|BB|AB|CT (6) 0.49 ± 0.11		AA|BB|AB|CT (6) 0.80 ± 0.35

The primers and genotyping identification method used in this study were suggested by Andreas et al. [14] (GHR), [15] (GHRH), [16] (PIT1), and [17] (STAT5A). The polymerase chain reaction (PCR) analysis proceeded by admixing the DNA sample (5–50 ng/μl), primers (200 ng/μl), PCR Master Mix (Kapa Taq ReadyMix), and H_2_O (12 μl final volume). The PCR process was set in 35 cycles with a specific temperature for certain primers to anneal (51°C–59°C). The *Alu*I, *Hae*III, *Hinf*I, and *Ava*I restriction enzymes (New England Biolabs) were used to identify the variation in the GHR, GHRH, PIT1, and STAT5A genes, respectively. Agarose gel electrophoresis (2%) was used to visualize the RFLP product.

The analysis of allele frequencies and heterozygosity was calculated using CONVERT [18] and CERVUS [19]. The evaluation of the genotype associations with growth performance indicators and DP was calculated using a general linear model implemented in Minitab v.14. Principal component analysis (PCA), including FactoMineR [20] and the factoextra package [21] in R 4.0.4, was performed to decrease the number of dimensions in the datasets, enhancing comprehensibility while reducing information loss [22].

## Results and Discussion

### Gene polymorphism

Polymorphism in the SO cattle GHR, GHRH, PIT1, and STAT5A genes in this study was illustrated by the different sizes and numbers of bands visualized in the electrophoresis system, known as the polymerase chain reaction-restriction fragment length polymorphism (PCR-RFLP) method. The PCR-RFLP method is still widely useful to identify the genotype of certain genes [23]. Based on the genotyping analysis results, the GHR, GHRH, PIT1, and STAT5A genes in this study showed polymorphism (Table 1). The polymorphism information of these genes in certain cattle breeds, especially SO cattle, is very important to provide an alternative breeding program in the future.

Based on the allele frequency information, the A allele (GHR and GHRH) and the B allele (PIT1) were higher than the other alleles (the value was more than 0.80). Meanwhile, the frequency of the T allele was lower than the C allele in the STAT5A gene (Table 1). In addition, our study also found similar findings from Hartati et al. [24] that the B allele is common in SO and Grati-Ongole grade cattle. Based on the polymorphism information content (PIC) value, the four genes used in this study had a low-to-moderate value. The highest PIC value in this study was 0.38 (STAT5A gene). The low PIC value in the GHR, GHRH, and PIT1 genes was to the allele frequency value that showed an unbalanced condition. In addition, the Ho value in this study was also low in all observed genes (except the STAT5A gene), which indicates the heterozygosity in the SO cattle is relatively low. Assortative mating and inbreeding could potentially be the cause [25]. The intense selection or the limited number of sires, as reported by Agung et al. [26], may have contributed to the low Ho and PIC values in the SO cattle population.

### PCA, growth, and DP analysis

**Figure 1.**
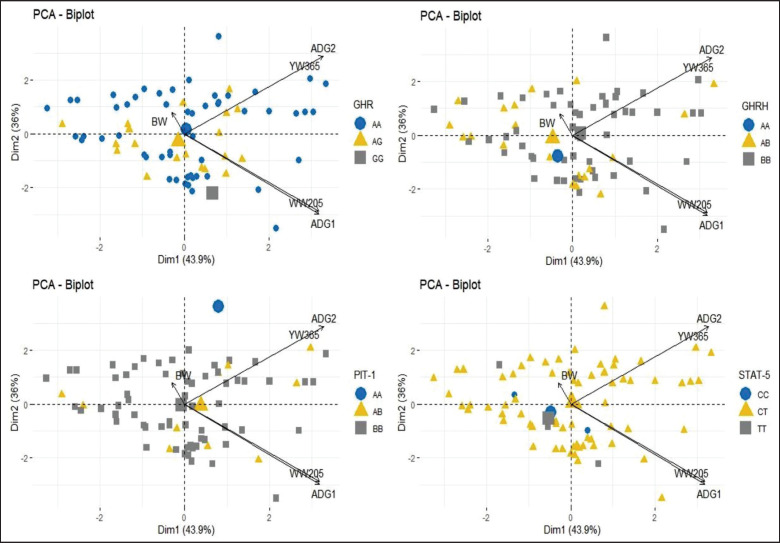
Principle component analysis—Biplot of growth traits and genotype.

All observed genes in this study did not affect growth traits (Tables 2 and 3). PCA is a multidimensional analysis looking at the distribution of data with many variables. Based on this analysis, WW205, ADG^1^, YW365, and ADG^2^ were affected by the AA genotype of the GHR gene (Fig. 1). The BW in Figure 1 shows a small variation, as shown by the short outline. The GHRH gene with the BB genotype influences WW205, ADG^1^, YW365, and ADG^2^.

The A allele in the GHRH gene has a positive effect. The quadrant of WW205, ADG1, YW365, and ADG2 displays the AB genotype. In the PIT-1 gene, the BW variable quadrant contained the BB genotype, which was more dominant, as well as WW205, ADG^1^, YW365, and ADG2 variables. In the STAT5A gene, the CT genotype predominates in the studied population in quadrants of the variables WW205, ADG^1^, YW365, and ADG^2^. In the STAT5A gene, the CT genotype predominates in the studied population in quadrants of the variables WW205, ADG^1^, YW365, and ADG^2^. However, based on PCA, it can be seen that environmental factors play a higher role because the genotypes obtained are spread across all quadrants.

The GHR gene polymorphism was reported to not affect the BW of Ongole grade cattle [27], but it was associated with adult weight in Pasudan cattle [28]. Furthermore, the GHR gene polymorphism showed significant effects on growth traits in Anatolian black cattle [29]. Meanwhile, a previous study reported that the PIT1 polymorphism did not affect the body weight of Pasundan cattle [30].

**Table 4.** Statistical analysis result of DP in the SO cattle based on genotypes.

Locus	Genotype	*N*	Mean (%) ± SD
GHR	AA	146	52.21 ± 4.44^a^
	AG	49	51.16 ± 5.75^ab^
	GG	5	46.93 ± 1.76^b^
GHRH	AA	6	54.79 ± 3.86^a^
	AB	62	51.39 ± 4.88^a^
	BB	132	51.89 ± 4.80^a^
PIT1	AA	2	54.66 ± 0.88^a^
	AB	25	50.51 ± 4.99^a^
	BB	173	51.98 ± 4.79^a^
STAT5A	CC	18	56.85 ± 3.35^a^
	CT	178	51.27 ± 4.68^b^
	TT	4	53.11 ± 3.59^ab^

*n =* number of individuals genotyped; SD=standard deviation; different superscript letters differ significantly (*p* < 0.05); significance test was conducted in each locus.

**Table 5.** Effect of genotype combinations of two locus on DP of SO cattle (*n =* 200).

Locus	Genotype combination (*n*)	Mean (%) ± SD	Locus	Genotype combination (*n*)	Mean (%) ± SD
GHR|GHRH	AA|AA (4)	54.26 ± 4.56^a^	GHR | PIT1	AA|AB (20)	51.61 ± 4.51^a^
	AA|AB (49)	51.99 ± 4.67^a^		AA|BB (125)	52.30 ± 4.45^a^
	AA|BB (93)	52.24 ± 4.33^a^		AG|AB (4)	46.46 ± 5.41^a^
	AG|AB (12)	49.50 ± 5.23^a^		AG|BB (44)	51.50 ± 5.68^a^
	AG|BB (35)	51.46 ± 5.93^a^		GG|BB (4)	47.47 ± 1.47^a^
	GG|BB (4)	47.47 ± 1.47^a^			
					
GHR|STAT5A	AA|CC (14)	56.57 ± 3.34^a^	GHRH|PIT1	AA|BB (6)	54.79 ± 3.86^a^
	AA|CT (130)	51.74 ± 4.29^bd^		AB|AB (10)	49.03 ± 5.06^a^
	AG|CC (4)	57.85 ± 3.64^ad^		AB|BB (52)	51.85 ± 4.76^a^
	AG|CT (43)	50.42 ± 5.63^bc^		BB|AB (15)	51.50 ± 4.85^a^
	GG|CT (5)	46.93 ± 1.76^bc^		BB|BB (115)	51.89 ± 4.84^a^
					
PIT1|STAT5A	AB|CT (22)	49.67 ± 4.58^a^	GHRH|STAT5A	AA|CT (4)	53.81 ± 3.89^ab^
	BB|CC (15)	56.89 ± 3.41^b^		AB|CT (59)	51.18 ± 4.83^a^
	BB|CT (154)	51.47 ± 4.68^a^		BB|CC (14)	56.90 ± 3.24^b^
	BB|TT (4)	53.11 ± 3.59^ab^		BB|CT (115)	51.26 ± 4.63^a^

*n =* number of individuals genotyped; SD=standard deviation; different superscript letters differ significantly (*p* < 0.05); genotype combination with n < 4 were not shown and not included in the analysis; significance test was conducted in each locus.

**Table 6.** Effect of genotype combinations of three and four locus on DP of SO cattle (*n =* 200).

Locus	Genotype combination (*n*)	Mean (%) ± SD	Locus	Genotype combination (*n*)	Mean (%) ± SD
GHR|GHRH|PIT1	AA|AA|BB (4)	54.26 ± 4.56^a^	GHR|GHRH|STAT5A	AA|AB|CT (46)	51.75 ± 4.62^ad^
	AA|AB|AB (7)	50.80 ± 4.63^a^		AA|BB|CC (10)	56.52 ± 3.20^e^
	AA|AB|BB (42)	52.19 ± 4.70^a^		AA|BB|CT (81)	51.71 ± 4.17^ac^
	AA|BB|AB (13)	52.05 ± 4.57^a^		AG|AB|CT (12)	49.50 ± 5.23^acd^
	AA|BB|BB (79)	52.25 ± 4.34^a^		AG|BB|CC (4)	57.85 ± 3.64^de^
	AG|AB|BB (10)	50.41 ± 4.98^a^		AG|BB|CT (30)	50.54 ± 5.76^ac^
	AG|BB|BB (32)	51.56 ± 5.97^a^		GG|BB|CT (4)	47.47 ± 1.47^ac^
	GG|BB|BB (4)	47.47 ± 1.47^a^			
GHR |PIT1|STAT5A	AA|AB|CT (18)	50.84 ± 4.00^a^	GHRH|PIT|STAT5A	AA|BB|CT (4)	53.81 ± 3.89^b^
	AA|BB|CC (12)	56.25 ± 3.43^b^		AB|AB|CT (10)	49.03 ± 5.06^ab^
	AA|BB|CT (111)	51.87 ± 4.35^ac^		AB|BB|CT (49)	51.61 ± 4.71^b^
	AG|BB|CT (39)	50.77 ± 5.52^ac^		BB|AB|CT (12)	50.21 ± 4.28^b^
	GG|BB|CT (4)	47.47 ± 1.47^ac^		BB|BB|CC (11)	56.96 ± 3.30^a^
				BB|BB|CT (101)	51.31 ± 4.70^b^
GHR|GHRH| PIT1|STAT5A	AA|AB|AB|CT (7)	50.80 ± 4.63^a^			
	AA|AB|BB|CT (39)	51.92 ± 4.66^a^			
	AA|BB|AB|CT (11)	50.87 ± 3.79^a^			
	AA|BB|BB|CC (8)	56.02 ± 3.26^a^			
	AA|BB|BB|CT (69)	51.81 ± 4.26^a^			
	AG|AB|BB|CT (10)	50.41 ± 4.98^a^			
	AG|BB|BB|CT (28)	50.64 ± 5.7^a^			
	GG|BB|BB|CT (4)	47.47 ± 1.47^a^			

*n =* number of individuals genotyped; SD=standard deviation; different superscript letters differ significantly (*p* < 0.05); genotype combination with n < 4 were not shown and not included in the analysis; significance test was conducted in each locus.

The statistical analysis (Table 4) showed that the SO cattle population with the CC genotype (STAT5A) had the highest DP value. The DP parameter and the genotypes of the GHR gene and the STAT5A gene showed a strong correlation (*p* < 0.05). On the other hand, no correlation (*p* < 0.05) was found between the GHRH and PIT1 genotypes and the DP parameter.

The genotype combination of two loci (Table 5) revealed that the highest DP value was found in the SO cattle population with AG|CC genotype (GHR|STAT5A combination) (57.85%) and the lowest DP value was found in the SO cattle population with AG|AB genotype (GHR|PIT1 combination) (46.46%). There were several genotype combinations that had significant effects on the DP value. The GHR|STAT5A, PIT1|STAT5A, and GHRH|STAT5A combination had a significant effect on the DP value. The GHR|STAT5A locus combination was shown to have a better DP value (especially the AG|CC genotype) than the other locus combination.

Based on the statistical analysis results with three locus combinations (Table 6), the highest DP value was found in the SO cattle population with AG|BB|CC genotype (GHR|GHRH|STAT5A combination) (57.85%), and all genotype combinations were associated significantly with the DP value (*p* < 0.05) except the GHR|GHRH|PIT1 locus. In addition, based on four locus combinations, the highest DP value was 56.02% (AA|BB|BB|CC genotype), and the lowest was 47.47%. There was no association (*p* > 0.05) between the four locus combinations and the DP value.

STAT5A has the highest effect on BW (Genotype TT) and DP (Genotype CC) (Table 7). On the other hand, PIT1 has the highest effect on WW205, YW365, ADG^1^, and ADG^2^. Table 7 also shows that the use of three SNPs on three genes is sufficient to perform marker-assisted selection. We can use genotypes AA|BB|AB (GHR|GHRH|PIT1) for WW205, YW365, ADG^1^, and ADG^2^. For BW selection, we can use Genotype AA|AB|BB (GHR|GHRH|PIT1). Furthermore, DP selection can use GHR|GHRH|STAT5A (Genotype AG|BB|CC). In this study, the frequency of STAT5A heterozygote animals was high. Meanwhile, the homozygote CC animals have the highest DP trait. Therefore, assortative mating in the heterozygote population can be performed to produce 50% of homozygote CC offspring. However, further validation of all genetic markers in this study (GHR, GHRH, PIT-1, and STAT5A) is still needed to establish a more accurate MAS in the SO cattle breeding programs.

## Conclusion

All observed genes in this study were polymorphic but did not have a significant effect on the body weight parameter. The GHR and STAT5A genes can be proposed as genetic markers for the high DP trait in SO cattle (*Bos indicus*).

**Table 7.** The highest effect of genotype combinations on growth traits of SO cattle.

Parameter	Locus	Genotype	Mean ± SD
BW (kg)	STAT5A	TT	27.67 ± 2.08
	GHR|GHRH	AA|AB	28.50 ± 4.40
	GHR|GHRH|PIT1	AA|AB|BB	28.75 ± 4.73
	GHR|GHRH|PIT1|STAT5A	AA|AB|BB|CT	29.00 ± 4.88
WW_205_ (kg)	PIT1	AB	115.08 ± 26.97
	GHRH|PIT1	BB|AB	124.40 ± 17.47
	GHR|GHRH|PIT1	AA|BB|AB	126.07 ± 18.52
	GHR|GHRH|PIT1|STAT5A	AA|BB|AB|CT	126.07 ± 18.52
YW_365_ (kg)	PIT1	AB	259.50 ± 129.50
	AA|AB	GHR|PIT1	285.30 ± 131.70
	GHR|GHRH|PIT1	AA|BB|AB	292.30 ± 125.90
	GHR|GHRH|PIT1|STAT5A	AA|BB|AB|CT	292.30 ± 125.90
ADG^1^ (kg/day)	PIT1	AB	0.44 ± 0.14
	GHRH|PIT1	BB|AB	0.49 ± 0.10
	GHR|GHRH|PIT1	AA|BB|AB	0.49 ± 0.11
	GHR|GHRH|PIT1|STAT5A	AA|BB|AB|CT	0.49 ± 0.11
ADG^2^ (kg/day)	PIT1	AB	0.71 ± 0.36
	GHR|PIT1	AA|AB	0.78 ± 0.36
	GHR|GHRH|PIT1	AA|BB|AB	0.80 ± 0.35
	GHR|GHRH|PIT1|STAT5A	AA|BB|AB|CT	0.80 ± 0.35
DP (%)	STAT5A	CC	56.85 ± 3.35
	GHR|STAT5A	AG|CC	57.85 ± 3.64
	GHR|GHRH|STAT5A	AG|BB|CC	57.85 ± 3.64
	GHR|GHRH|PIT1|STAT5A	AA|BB|BB|CC	56.02 ± 3.26

BW=birth weight (kg); WW_205_=weaning weight at 205 days of age (kg); ADG^1^=weaning average daily gain (kg/day); YW_365_=yearling weight at 365 days of age (kg); ADG^2^=post weaning average daily gain (kg/day).

## List of Abbreviations

DNA, Deoxyribonucleic acid; GH, Growth hormone; GHR, Growth hormone receptor; GHRH, Growth hormone releasing hormone; GLM, General linear model; IGF-1, Insulin-like growth factor 1; MAS, Marker assisted selection; MC4R, Melanocortin‐4 receptor; PCA, Principal component analysis; PCR, Polymerase chain reaction; PT.KAR, Karya Anugerah Rumpin Corporation; PIT-1, Pituitary-specific transcription factor-1; RFLP, Restriction fragment length polymorphism; STAT5A, Signal transducer and activator of transcription 5A; ng, Nanogram; μl, microliter; °C, the degree Celsius.
